# MicroRNAs and cardiac sarcoplasmic reticulum calcium ATPase-2 in human myocardial infarction: expression and bioinformatic analysis

**DOI:** 10.1186/1471-2164-13-552

**Published:** 2012-10-15

**Authors:** Emanuela Boštjančič, Nina Zidar, Damjan Glavač

**Affiliations:** 1 Department of Molecular Genetics, Institute of Pathology, Korytkova 2, Faculty of Medicine, Ljubljana, Slovenia

**Keywords:** Human myocardial infarction, Expression, SERCA2, miRNA, Bioinformatics

## Abstract

**Background:**

Cardiac sarco(endo)plasmic reticulum calcium ATPase-2 (SERCA2) plays one of the central roles in myocardial contractility. Both, SERCA2 mRNA and protein are reduced in myocardial infarction (MI), but the correlation has not been always observed. MicroRNAs (miRNAs) act by targeting 3'-UTR mRNA, causing translational repression in physiological and pathological conditions, including cardiovascular diseases. One of the aims of our study was to identify miRNAs that could influence SERCA2 expression in human MI.

**Results:**

The protein SERCA2 was decreased and 43 miRNAs were deregulated in infarcted myocardium compared to corresponding remote myocardium, analyzed by western blot and microRNA microarrays, respectively. All the samples were stored as FFPE tissue and in RNA*later*. miRNAs binding prediction to SERCA2 including four prediction algorithms (TargetScan, PicTar, miRanda and mirTarget2) identified 213 putative miRNAs. TAM and miRNApath annotation of deregulated miRNAs identified 18 functional and 21 diseased states related to heart diseases, and association of the half of the deregulated miRNAs to SERCA2. Free-energy of binding and flanking regions (RNA22, RNAfold) was calculated for 10 up-regulated miRNAs from microarray analysis (*miR-122, miR-320a/b/c/d, miR-574-3p/-5p, miR-199a, miR-140,* and *miR-483*), and nine miRNAs deregulated from microarray analysis were used for validation with qPCR (miR*-21, miR-122, miR-126, miR-1, miR-133, miR-125a/b,* and *miR-98*). Based on qPCR results, the comparison between FFPE and RNA*later* stored tissue samples, between Sybr Green and TaqMan approaches, as well as between different reference genes were also performed.

**Conclusion:**

Combing all the results, we identified certain miRNAs as potential regulators of SERCA2; however, further functional studies are needed for verification. Using qPCR, we confirmed deregulation of nine miRNAs in human MI, and show that qPCR normalization strategy is important for the outcome of miRNA expression analysis in human MI.

## Background

A growing body of evidence has revealed that dysfunction of 3'- and 5'-untranslated regions (UTRs) is linked to the pathophysiology of variety of diseases. Both UTRs are important as regulatory elements of mRNA translation [1]. One of the negative regulators of mRNA translation are small non-coding RNAs, microRNAs (miRNAs) that bind to 3'-UTR of target mRNA. By binding to target mRNA, miRNAs have been found to regulate a variety of physiological functions and contribute to various diseases, including ischemic heart diseases [2]. It has been suggested that some parts of the conserved regions of the 3'-UTRs of SERCA2 might represent post-transcription binding sites for miRNAs [3].

SERCA2 is muscle-specific sarco(endo)plasmic reticulum calcium ATPase-2, which promotes cardiac relaxation and contractility. During every heartbeat, a pulse of calcium is released from the sarcoplasmic reticulum (SR) into the cytoplasm of cardiac muscle cells through a calcium release channel (RyR, ryanodine receptor), thus triggering muscle contraction. During relaxation, SERCA2 re-sequesters the calcium back into the internal sarcoplasmic reticulum storage pool, thus priming the next release of calcium [4]. SERCA2 pre-mRNA is edited depending on the cellular type. Due to different alternative splicing, the last exons of SERCA2 pre-mRNA produce different mRNAs, which differ at the 3´-UTR regions and encode for SERCA2a, SERCA2b and SERCA2c isoforms. In the heart, SERCA is mainly represented by the SERCA2a isoform, which is expressed in the heart and slow-twitch skeletal muscle [3,5]. SERCA2b, ubiquitously expressed in all tissues, may at least partially replace SERCA2a function [6].

Decreased expression of the SERCA2 is one of the key features of cardiac myocyte dysfunction in both experimental and human heart failure as well as in myocardial infarction MI [3,4,6,7]. It is believed that this decrease contributes to abnormal contractility in post-infarction rat myocytes and that increased expression of SERCA2 could improve myocardial contractility in cardiovascular diseases [8,9]. It has been shown that the main regulators of SERCA2 protein and cardiac contractility are phospholamban (PLN) in the ventricles and sarcolipin (SLN) in the atria [3]. A simultaneous decrease in SERCA2 mRNA and protein levels is not always observed [7], and there remain gaps in understanding regulation of SERCA2 by PLN and SLN in the heart [4].

Since functionally critical genes that are spatially expressed are stringently regulated by miRNAs [10], was the aim of our study to test for miRNA and SERCA2 expression in infarcted myocardium compared to corresponding remote myocardium of patients with MI, and defined by target prediction algorithms those miRNAs with a potential influence on SERCA2 mRNA 3'-UTR. Differentially expressed miRNAs were further investigated using bioinformatic approaches as possible regulators of SERCA2 protein regulators (PLN, SLN). We also compared the *miR-26b* and *RNU6B* as reference genes (RGs), both of which were used as endogenous controls in our previous study [11]. Two different approaches were used (TaqMan and Sybr Green), as well as two different types of tissues (RNA*later*, FFPE).

## Results

### Western blot

**Figure 1:**
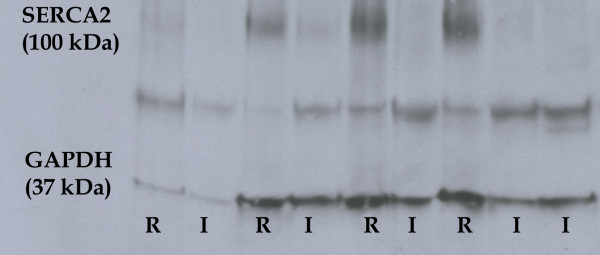
**Western blot results of SERCA2 expression in the infarcted tissue and corresponding remote myocardium.** SERCA2 (~100 kDa) is under-expressed in the infarcted tissue when compared to corresponding remote myocardium. GAPDH (~37 kDa), used as internal control, showed similar expression in infarcted and remote myocardium of patients with myocardial infarction. Legend: I, infarcted tissue, R, corresponding remote myocardium.

In 4 out of 6 tested samples of infarcted tissues, it was possible to extract enough proteins in a defined volume to get comparable result using western blot (20 μg). In all four tested tissue samples we observed decreased SERCA2 protein expression in the infarcted tissue (I) when compared to corresponding remote (R) myocardium. The GAPDH protein, used as endogenous control, showed no change in the expression between infarcted tissue and corresponding remote myocardium. The results are summarized in Figure 1.

### MicroRNA microarray results

**Figure 2:**
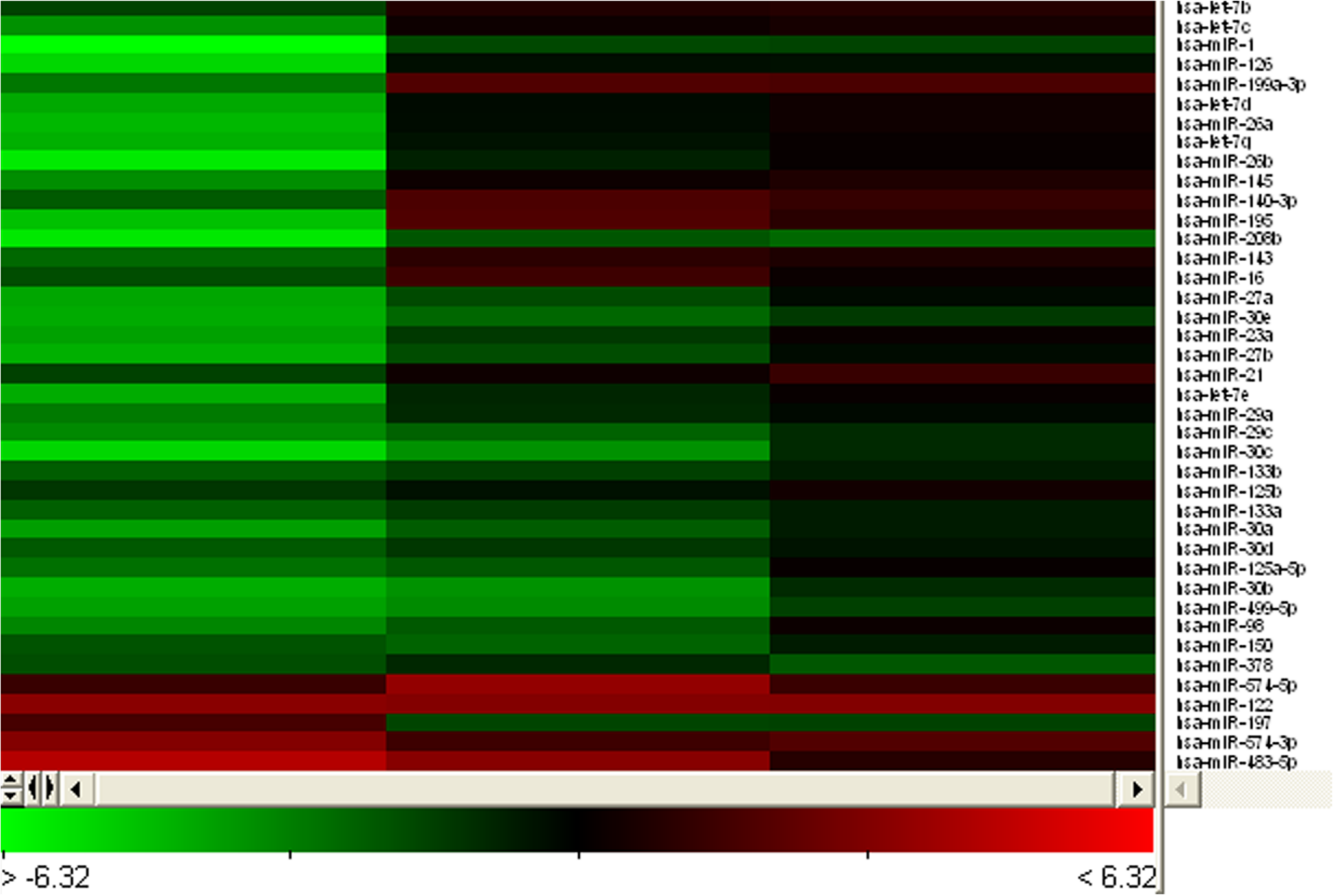
**Hierarchical clustering analysis of the log**_**2**_**value of differentially expressed miRNAs in myocardial infarction.** Dual colour experiments and swap-dye were performed, using Cy3 and Cy5 to compare miRNAs expression in infarcted tissue and corresponding remote myocardium. Statistical analysis and hierarchical clustering was performed using Acuity 4.0 analytical software. The results are displayed in a heat map. The legend on the right indicates the miRNA shown in the corresponding row, all of which are human specific. The bar code on the bottom shows the colour scale of the log_2_ value. Each column represents data from one microarray. The heat map was constructed of 43 miRNAs dysregulated in all of the microarrays performed.

Microarray data are deposited to the Gene Expression Omnibus public depository (GSE17304). MicroRNA microarray expression analysis revealed 43 differentially expressed miRNAs in infarcted tissue when compared to corresponding remote myocardium. Ten of the 43 differentially expressed miRNAs showed up-regulation. The results are summarized in Table 1. Unsupervised hierarchical clustering was performed using all miRNAs being differentially expressed in microarray analysis. The results are summarized in Figure 2. Since SERCA2 protein showed down-regulation in infarcted tissue compared to corresponding remote myocardium, the resulting 10 miRNAs, which showed up-regulation, were subject to further bioinformatics analysis.

**Table 1:** Differentially expressed miRNAs in infarcted tissue compared to corresponding remote myocardium, their classification (from miRBase) and prediction of targeting SERCA2 (PicTar, TargetScan, miRanda, mirTarget2)

**miRNA**	**average log**_**2**_	**average log**_2_*	**predicted**	**miRNA Gene family**	**Clustered** **(chr: miR; location)**
*let-7b*	0.04	−0.32	Yes	let-7	22: let-7a-3, miR-4763; intronic
*let-7c*	−0.87	−0.88	Yes	let-7	21: miR-99a; intronic
*let-7d*	−1.34	−1.33	Yes	let-7	9: let-7a-1, let-7f-1; intronic
*let-7g*	−1.50	−1.67	Yes	let-7	3: as single gene; intronic
*miR-1*	−3.28	−1.89	No	miR-1	18: miR-133a-1; intronic
					20: miR-133a-2; intronic
*miR-122*	3.28	ND	No	miR-122	18: miR-3591; intergenic
*miR-125a-5p*	−1.53	−1.46	No	miR-125	19: let-7e, miR-99b; intronic
*miR-125b*	−0.44	−0.47	No	miR-125	11: as single gene; intronic
*miR-126*	−2.00	−0.50	No	miR-126	9: as single gene; intronic
*miR-133a*	−1.52	ND	No	miR-133	18: miR-1-2; intronic
					20: miR-1-1; intronic
*miR-133b*	−1.53	ND	No	miR-133	6: miR-206; intronic
*miR-140-3p*	0.32	ND	No	miR-140	16: as single gene; intronic
*miR-143*	−0.26	ND	No	miR-143	5: miR-145; intergenic
*miR-145*	−0.79	ND	No	miR-145	5: miR-143; intergenic
*miR-150*	−1.63	−1.60	No	miR-150	19: as single gene; intergenic
*miR-16*	−0.03	ND	No	miR-15	13: miR-15a; intronic
					3: miR-15a; intronic
*miR-195*	−0.51	−1.37	Yes	miR-15	17: miR-497; intronic
*miR-197*	−0.57	ND	No	miR-197	1: as single gene; intergenic
*miR-199a-3p*	0.39	ND	No	miR-199	19: as single gene; intronic
					1: miR-199a-2, miR-3120, miR-214; intronic
*miR-19b*	−2.36	ND	Yes	miR-19	13: miR-17, miR-18a, miR-19a, miR-20a, miR-92a-1; intergenic
					X: miR-106a, miR-18b, miR-20b, miR-92a-2, miR-363; intergenic
*miR-21*	−0.05	ND	No	miR-21	17: as single gene; intergenic
*miR-23a*	−1.71	−0.20	No	miR-23	19: miR-27a, miR-24-2; intergenic
*miR-26a*	−1.50	−0.54	No	miR-26	3: as single gene; intronic
					12: as single gene; intronic
*miR-27a*	−1.97	−1.60	No	miR-27	19: miR-23a, miR-24-2; intergenic
*miR-27b*	−2.18	−1.19	No	miR-27	9: miR-23b, miR-24-1, miR-3074; intronic
*miR-29a*	−1.40	ND	Yes	miR-29	7: miR-29b-1; intergenic
*miR-29c*	−2.28	ND	No	miR-29	1: miR-29b-2; intergenic
*miR-30a*	−2.23	−1.25	Yes	miR-30	6: as single gene; intronic
*miR-30b*	−3.03	−2.01	Yes	miR-30	8: miR-30d; intergenic
*miR-30c*	−2.70	−2.08	Yes	miR-30	1: miR-30e; intronic
					6: as single gene; intronic
*miR-30d*	−1.31	ND	Yes	miR-30	8: miR-30b; intergenic
*miR-30e*	−2.71	ND	Yes	miR-30	1: miR-30c-1; intronic
*miR-320a*	2.37	0.98	No	miR-320	8: as single gene; intergenic
*miR-320b*	2.24	0.98	No	miR-320	1: two copies as single gene; one intronic
					and one intergenic
*miR-320c*	2.27	0.98	No	miR-320	18: two copies as single gene; intronic
*miR-320d*	2.13	0.98	No	miR-320	13: as single gene; intergenic
					X: as single gene; intergenic
*miR-378*	−1.66	ND	No	miR-378	5. as single gene; intronic
*miR-483-5p*	2.92	ND	No	miR-483	11: as single gene; intronic
*miR-499-5p*	−2.94	−3.36	Yes	miR-499	20: miR-499a; intronic
*miR-574-3p*	2.25	2.46	Yes	miR-574	4: as single gene; intronic
*miR-574-5p*	2.17	3.18	No	miR-574	
*miR-98*	−1.76	−3.10	Yes	let-7	X: let-7f; intronic

Legend: *, results from previous microarray study [11], where infarcted tissue was compared to healthy adult hearts (independent groups of samples); ND, not determinate; chr, chromosome.

We further compared our results to our previous study based on microarray analysis [11], where the miRNA expression in infarcted tissue was calculated relative to the miRNA expression in healthy human hearts (independent groups of samples). Of 43 differentially expressed miRNAs in infarcted tissue compared to remote myocardium, 20 miRNAs showed similar expression also when infarcted tissue was compared to healthy human hearts, 4 were specifically differentially expressed in the present study, and 19 showed higher difference in expression in the present study. The results are summarized in Table 1.

### Bioinformatic analysis of miRNAs

#### miRNA prediction

Using four different available prediction algorithms, namely TargetScan [12], PicTar [13], mirTarget2 included in miRDB [14], and miRanda [15] included in MicroCosm Targets (former miRBase) [16] and in microrna.org [17], 213 miRNAs were predicted for binding to 3'-UTR mRNA SERCA2. However, target prediction programs did not distinguish between isoforms SERCA2a and SERCA2b. Of 213 predicted miRNAs, 136 corresponded to an isoform SERCA2b and 74 to an isoform SERCA2a. Significantly, TargetScan5.1 gave no results for isoform SERCA2b, and mirTarget2 predicted only one miRNAs to target SERCA2b. In the case of SERCA2a, all of the used algorithms predicted miRNAs (Additional file 1: Table S1). Of all 213 miRNAs predicted to target SERCA2, 15 were differentially expressed in human MI (Table 1).

#### Free-energy analysis

It has been recently demonstrated with the majority of miRNA-validated targets that miRNAs preferentially bind to 3'-UTR sites that do not have complex secondary structures and are located in accessible regions of the mRNA based on favourable thermodynamics. Ten miRNAs were used for further *in silico* validation: one predicted by using above programs with elevated expression in microarray analysis (*hsa-miR-574-3p;* miRBase accession number: MIMAT0003239) and nine up-regulated in microarray analysis but not predicted by the algorithms used *(hsa-miR-122, hsa-miR-199a-3p, hsa-miR-140-3p, hsa-miR-320a, hsa-miR-320b, hsa-miR-320c, hsa-miR-320d, hsa-miR-483-5p, hsa-miR-574-5p;* miRBase accession numbers: MIMAT0000421, MIMAT0000232, MIMAT0004597, MIMAT0000510, MIMAT0005792, MIMAT0005793, MIMAT0006764, MIMAT0004761, MIMAT0004795, respectively). Although is believed that SERCA2a is the major isoform in the heart, our further analysis focused on both isoform SERCA2a and SERCA2b, since primary antibody used in our western blot analysis did not distinguish between isoform SERCA2a and SERCA2b. All results for SERCA2a and SERCA2b are summarized in Table 2 and Table 3, respectively.

**Table 2:** miRNAs with predicted influence on SERCA2a expression

**MicroRNA**	**ΔG binding (Kcal mol**^**-1**^**)**	**ΔG 70 nt flanking 3’ of predicted binding site (Kcal mol**^**-1**^**)**	**ΔG 70 nt flanking 5’ of predicted binding site (Kcal mol**^**-1**^**)**	**Predicted binding position in 3’-UTR SERCA2a**	**Perfect SERCA2a complimentarity to “seed” region**
*miR-122*	−22.0	−14.9	−11.2	671-692	partially (1–6)
*miR-199a-3p*	−32.1	−9.1	−14.1	76-97	no
	−30.7	−11.1	−6.9	535-556	no
	−24.3	−9.2	−6.9	705-726	no
	−22.0	−7.6	−11.3	259-280	no
	−20.5	−10.1	−6.6	26-47	no
*miR-320a*	−24.6	−10.9	−7.1	117-138	partially (1–5, 7–12)
	−21.7	−6.9	−11.1	536-557	partially (1–2, 4–8)
	−24.8	−12.3	−11.4	505-526	partially (1–4, 7–8)
	−25.5	−6.9	−12.9	229-250	no
	−20.5	−8.5	−5.3	711-732	no
*miR-320b*	−24.6	−10.9	−7.1	117-138	partially (1–5, 7–12)
	−21.7	−6.9	−11.1	536-557	partially (1–2, 4–8)
	−25.8	−12.3	−11.4	505-526	partially (1–4, 7–8)
	−25.5	−6.9	−12.9	229-250	no
*miR-320c*	−23.2	−6.9	−12.9	230-249	no
	−23.3	−12.3	−11.4	507-526	partially (1–4, 7–8)
	−19.5	−7.7	−5.3	713-732	no
*miR-320d*	−24.4	−10.1	−7.1	120-138	partially (1–5, 7–13)
	−19.4	−6.9	−11.1	539-557	partially (1–2, 4–8)
	−22.8	−6.9	−12.9	231-249	no
	−23.1	−12.3	−11.4	508-526	partially (1–4, 7–8)
	−19.2	−6.4	−5.3	714-732	no
*miR-483-5p*	−24.7	−9.8	−6.8	122-143	no
	−24.1	−7.6	−6.3	708-729	no
*miR-574-3p*	−27.8	−8.6	−5.3	725-746	yes
	−32.5	−7.3	−9.7	37-58	partially (2–5, 7–13)
*miR-574-5p*	−22.7	−7.3	−11.0	465-487	yes
	−25.6	−14.5	−14.1	664-686	no
	−30.2	−12.5	−0.3	740-762	no
	−22.9	−8.0	−12.3	413-435	no
	−21.6	−7.8	−11.1	364-386	no
*miR-140-3p*	−19.1	−11.3	0.3	746-766	partially (1–4)
	−20.2	−7.3	−9.7	38-58	yes
	−24.2	−7.7	−5.3	713-733	yes

**Table 3:** miRNAs with predicted influence on SERCA2b expression

**MicroRNA**	**ΔG binding (Kcal mol**^**-1**^**)**	**ΔG 70 nt flanking 3’ of predicted binding site (Kcal mol**^**-1**^**)**	**ΔG 70 nt flanking 5’ of predicted binding site (Kcal mol**^**-1**^**)**	**Predicted binding position in 3’-UTR SERCA2b**	**Perfect SERCA2b complimentarity to “seed” region**
*miR-122*	−24.9	−9.9	−8.7	313-334	no
	−23.7	−3.0	−8.2	788-809	no
	−21.5	−0.2	−9.5	496-517	no
	−19.3	−8.2	−7.5	253-274	no
	−19.3	−3.5	−5.2	476-497	no
	−19.3	−6.3	−9.0	458-479	no
	−18.1	−4.3	−8.3	151-172	no
*miR-199a-3p*	−22.0	−4.9	−9.5	380-401	yes
	−21.0	−3.1	−11.1	487-508	yes
	−18.6	−8.3	−3.9	59-80	yes
	−18.1	−7.5	−8.3	251-272	no
*miR-320a*	−21.2	−9.9	−9.3	405-426	no
	−21.8	−9.0	0.3	798-819	no
	−21.2	−4.7	−9.8	65-86	partially (1–4)
	−23.7	−8.2	−8.2	687-708	no
	−21.8	−9.1	−11.9	123-144	partially (1–6)
*miR-320b*	−21.2	−9.9	−9.3	405-426	no
	−21.8	−9.0	0.3	798-819	no
	−21.2	−4.7	−9.8	65-86	partially (1–4)
	−23.7	−8.2	−8.2	687-708	no
	−21.8	−9.1	−11.9	123-144	partially (1–6)
*miR-320c*	−19.1	−8.0	0.3	800-819	no
	−24.0	−12.4	−8.2	689-708	no
	−21.1	−11.4	−9.2	650-669	no
*miR-320d*	−23.4	−13.2	−8.2	690-708	no
	−24.3	−12.2	−9.0	709-727	partially (1–4, 6–7)
*miR-483-5p*	−23.8	−13.1	−9.0	705-726	no
	−21.8	−4.7	−8.5	63-84	no
	−22.7	−9.1	−9.3	121-142	no
	−20.3	−8.2	−3.0	789-810	Partially (1, 3–6, 8)
*miR-574-3p*	−22.9	−8.2	−6.5	347-368	No
	−21.2	−2.7	−11.3	96-117	no
*miR-574-5p*	−21.7	−8.6	−8.0	263-285	No
	−23.4	−4.9	−3.5	479-501	No
	−18.2	−3.3	−3.5	12-34	No
	−20.5	−9.0	0.3	797-819	Partially (1–5, 7)
*miR-140-3p*	−19.9	−8.2	−6.5	348-368	yes
	−20.5	−9.9	−3.5	484-504	No
	−19.7	−8.7	0.3	799-819	no

Using criteria postulated by Zhao et al. (2005) [18], we predicted binding sites in 3´-UTR of SERCA2b and SERCA2a mRNA for up-regulated miRNAs. Using RNA22 algorithm [19], some miRNAs were predicted to have over 10 potential binding sites either in SERCA2a or SERCA2b. In case of SERCA2b, 13 binding sites were predicted for *hsa-miR-320a/b*, 12 for *hsa-miR-320c/d*, 15 for *hsa-miR-574-5p* and 20 for *hsa-miR-122*. In case of SERCA2a, 16 binding sites were predicted for *hsa-miR-574-5p*. We therefore presented those with the highest difference (at least 10 kcal/mol) between the potential binding site and the 70 nt flanking 3' and 5' of predicted binding site based on calculation using RNAfold [20]. Large proportion of binding sites is located in certain positions in 3'-UTRs of corresponding SERCA2a and SERCA2b. In SERCA2a, there are 7 “hot-spot” locations for miRNA binding and in SERCA2b there are 9 “hot-spot” locations. For most of predicted binding sites there is more than one predicted miRNA (cooperativity), and this is further attenuated by more than one binding site for one miRNA (multiplicity). Most of predicted miRNAs have at least one binding site with perfect complimentarity to seed region.

#### miRNA annotation and functional classification

Analysis using miRBase revealed that differentially expressed miRNAs belongs to 25 miRNA Gene family, and at least one gene copy of 25 differentially expressed miRNAs is clustered. Further, the 4 clusters are fully represented among differentially expressed miRNAs. The results are summarized in Table 1.

TAM tool [21] was used to annotate differentially expressed miRNAs in the mean of function and disease associations. Among 35 defined functions for differentially expressed miRNAs, 18 are related to heart diseases (Additional file 2: Table S2). Further, differentially expressed miRNAs are related to 136 different disease outcomes, 21 are related to heart diseases (Additional file 3: Table S3).

Finally, miRNApath [22] revealed that calcium signalling pathway includes 175 genes, and 468 miRNAs regulating these genes. Of these, 95 miRNAs are associated with SERCA2, 19 miRNAs with PLN, and 21 miRNAs with SLN. Of differentially expressed miRNAs in our study, 18 are according to miRNApath related to SERCA2, none is related to PLN, and three are related to SLN. In summary, only *miR-30a/e* and *miR-145* are differentially expressed and related to SERCA2 as well as to its regulator SLN (data not shown).

### Quantitative real-time PCR

Using two different qPCR technologies, we validated the expression of nine miRNAs. Sybr Green technology was used to validate: *miR-122*, which was specifically expressed on present microarrays compared to our previous study [11] and it showed the highest up-regulation; *miR-21* and *miR-126*, which are in addition to muscle-specific *miR-1* and *miR-133* the most common miRNAs involved in heart diseases; *miR-125a/b*, which are according to TAM tool involved in myocardial remodelling after MI. TaqMan based approach was used to validate *miR-98*, which was one of the few miRNAs overlapping target prediction and is according to TAM tool involved in hypertrophy; and *miR-1* and *miR-133a/b*, muscle-specific miRNAs.

In addition to microarray validation, the qPCR methodology was used to test several presumptions. First, *miR-26b* was tested as RG in comparison to *RNU6B*. Both were used as RGs in our previous study [11], using *miR-26b* as RG in TaqMan based approach and *RNU6B* as RG in Sybr Green approach. In present study, both were used in TaqMan as well as in Sybr Green technology. The expression of *RNU6B* showed relative stability in both approaches, as well as in RNA*later* and FFPE tissue samples. When the *RNU6B* expression using Sybr Green (performed on Rotor Gene Q) was compared to the results from same tissue from previous study (performed on ABI7900), the expression showed same stability, except that the Cq-values were higher in previous study for 2.38 ± 0.39. *miR-26b* showed similar expression to *RNU6B* in RNA*later* stored tissue (TaqMan or Sybr Green) as well as in FFPE samples (TaqMan), but it not seems to be suitable as RG, when validating FFPE using Sybr Green (SD is much higher when using *miR-26b* in comparison to *RNU6B*). The results are summarized in Table 4.

**Table 4:** Stability of reference genes in qPCR of tested samples

**Technology**	**TaqMan**						**Sybr Green**			
**RG tested**	***miR-26b***		***RNU6B***		***RNU48***		***miR-26b***		***RNU6B***	
Measurements	Cq	SD	Cq	SD	Cq	SD	Cq	SD	Cq	SD
FFPE	26.67	2.95	32.56	2.41	29.36	2.11	28.56	2.99	19.83	1.58
RNA*later*	23.63	0.91	30.24	1.27	25.71	0.96	24.46	1.21	14.66	1.22

Legend: RG, reference gene; Cq, tershold/quantification cycle; SD, standard deviation; FFPE, formalin fixed paraffin-embeded tissue; RNA*later*, RNA*later* stored samples.

Second, we compared Sybr Green and TaqMan approach. The results of *miR-26b* relatively to *RNU6B* were similar across the samples using either Sybr Green or TaqMan based approach, either FFPE or RNA*later* stored tissue samples (data not shown).

Third, the comparison between RNA*later* and FFPE tissue samples has been performed. The results are summarized in Table 5. Using both technologies (TaqMan and Sybr Green), we confirmed most of the microarray results using *RNU6B* as RG, as well as using *miR-26b* (only in case of TaqMan approach). Both was true for FFPE but not for RNA*later* stored samples. However, some discrepancies can be seen between RNA*later* and FFPE samples using Sybr Green approach. It can be also noted from Table 5 that in the case of Sybr Green, using *miR-26b* as RG for FFPE samples gives different results from using *RNU6B* as RG. *RNU6B* as RG with FFPE samples (Sybr Green) is in accordance to microarray results, and only expression of miR*-122* relatively to *miR-26b* in FFPE samples corresponds to microarray results (Sybr Green). In contrast, results from RNA*later* samples are similar between those that use *RNU6B* as RG and those that use *miR-26b* as RG.

**Table 5:** Ratios of differentially expressed miRNAs in infarcted tissue using TaqMan based and Sybr Green approach

	**TaqMan**				**Sybr Green**				
	***miR-1***	***miR-133a***	***miR-133b***	***miR-98***	***miR-21***	***miR-122***	***miR-125a-5p***	***miR-125b***	***miR-126***
FFPE *RNU48*	0.10	1.24	0.25	0.36	nd	nd	nd	nd	nd
RNA*later RNU48*	0.47	0.46	0.39	0.62	nd	nd	nd	nd	nd
FFPE *RNU6B*	0.11	0.68	0.29	0.41	0.59	1.71	0.49	0.44	0.29
RNA*later RNU6B*	0.22	0.22	0.18	0.29	1.65	0.18	1.04	1.18	0.99
FFPE *miR-26b*	0.20	1.25	0.53	0.74	5.03	14.52	4.21	3.73	2.49
RNA*later miR-26b*	0.78	0.77	0.64	1.02	2.64	0.29	1.67	1.90	1.59
microarray results	0.10	0.35	0.35	0.30	0.97	9.71	0.35	0.74	0.25

The ratios are calculated according to the efficiency corrected model [46]. Expression in remote myocardium is set at one, and expression in infarcted tissue is calculated as a proportion of the expression in corresponding remote myocardium. Further, three different genes were used as RGs. Legend: FFPE, formalin-fixed paraffin-embedded tissue; RNA*later*, samples stored in RNA*later*; *RNU48*, the small RNA *RNU48* is used as RG; *RNU6B*, the small RNA *RNU6B* is used as RG; *miR-26b*, the *miR-26b* is used as RG.

Statistical analysis revealed that *miR-1*, *miR-133a/b* and *miR-98* expression based on qPCR results is in accordance to microarray results, that are down-regulated in infracted compared to corresponding remote myocardium, but statistical significance is dependent of RG used (Table 5). In contrast, expression of *miR-1* and *miR-133a/b* is always in statistical significant correlation to each other not dependent of RG used (data not shown). The same is true for expression of *miR-125a-5p* and *miR-125b*. Expression of miRNAs (*miR-21, miR-125a/b, miR-122 and miR-126*) is dependent of RG and type of tissue. The expression of all five miRNAs was in accordance to microarray results in FFPE, when *RNU6B* was used as RG. In contrast, comparing *miR-26b* and *RNU6B* as RG, the different outcomes were detected in FFPE samples, but similar (and different from microarray study) in RNA*later* stored samples. As well as previously described [23] we observed superior sensitivity of qPCR over microarray analysis and did not observe significant variation potentially introduced by reverse transcription.

## Discussion

In our study, we showed a reduced expression of protein SERCA2 in infarcted tissue when compared to corresponding remote myocardium of patients with MI. These results are in accordance with previously demonstrated decrease in SERCA2 mRNA and protein levels in ischemic-reperfused heart and animal models of myocardial ischemia [7]. A decrease in content and activity of SERCA2 is also observed during ageing [4]. To eliminate misinterpretation of western blot results due to different ages of our patients, we did not compare SERCA2 in remote myocardium and infarcted tissue of patients with MI to healthy adult hearts. Since simultaneous decrease in SERCA2 mRNA and protein levels is not always observed [7], and there remain gaps in understanding regulation of SERCA2 by its protein regulators [4], we presumed that SERCA2 might be regulated by miRNAs. MicroRNA microarray expression analysis revealed 43 differently expressed miRNAs in infarcted tissue when compared to corresponding remote myocardium, 10 of these showing up-regulation (speculatively being involved in SERCA2 regulation). According to miRNApath analysis of calcium signalling pathway, approx. half of differentially expressed miRNAs are related to SERCA2 but not to its regulatory proteins. Compared to our previous study based on microarray analysis [11], where the miRNA expression in infracted tissue was calculated relative to the miRNA expression in healthy human hearts (independent groups of samples), only half of miRNAs showed similar expression in the present study, suggesting specifically expressed miRNAs in infracted tissue as well as in remote myocardium compared to healthy adult hearts. This is in accordance to recent findings that some miRNAs in the non-infarcted area might also participate in the pathophysiology response to MI [24].

SERCA2 has two major isoforms, SERCA2a and SERCA2b, both of which are expressed in heart from early developmental stages, although it is believed that SERCA2a is the major isoform expressed in the heart [5]. Using four different available prediction algorithms, 213 miRNAs were predicted for binding to 3'-UTR mRNA SERCA2; however, used algorithms did not distinguish between SERCA2 isoforms. Of predicted miRNAs, two times more corresponded to an isoform SERCA2b than to an isoform SERCA2a, and only approx. 10% were differentially expressed in infarcted tissue compared to remote myocardium. Finally, only one of predicted and differentially expressed was up-regulated (*miR-574-3p*).

*miR-574-3p* was also predicted to target ATP2A2 in our previous study, and has been elevated in infarcted compared to healthy adult hearts [11]. We therefore used this and other up-regulated miRNAs to search for potential binding using algorithm RNA22. The interaction of miRNA:mRNA usually occurs via non-strict base pairing, and the most important should be matching to the 1–8 nucleotides from the 5´ end of miRNA (“seed” region). miRNA-mRNA binding may also occur in other ways [25]. Some features of miRNA:mRNA binding have been implicated in bioinformatics and experimental approaches, but most of the target sites identified by available tools are based on seed region complimentarity, therefore might be biased against the one class of binding sites. Since the majority of a given mRNA sequence is highly structured and local RNA accessibility of the binding site may be a critical feature of miRNA target recognition [18], we determined the free-energy (ΔG) of the miRNA:mRNA binding. We further tested all 10 up-regulated miRNAs for free-energy of 70 nt of 5´ and 3´ of the predicted binding sites to test whether the predicted binding sites are located in a region of very high free energy, suggesting a locally accessible site. Complex RNA secondary structures may prevent miRNA/mRNA interactions and may have inhibitory effects on miRNA:mRNA interactions. Repression of mRNAs may be also increased by multiplicity (several binding sites in a transcript for a single miRNA) and cooperativity (several miRNAs bind to a single transcript) [25]. In line with that, several miRNAs were predicted for binding to the same positions of 3´-UTR SERCA2 (7 predicted miRNAs binding positions for SERCA2a, and 9 predicted miRNAs binding positions for SERCA2b), and for the majority of the predicted miRNAs there was more than one putative binding site. Most of predicted miRNAs have at least one binding site with perfect complimentarity to seed region.

As previously described, some of the up-regulated and *in silico* validated miRNAs have been already related to cardiovascular diseases [26]. In addition to *miR-122,* which was specifically expressed on present microarrays compared to our previous study [11] and is according to recent report down-regulated in plasma of patients with MI [27], *miR-574* and *miR-140* have not been yet described as involved in cardiovascular pathology. Other up-regulated and *in silico* validated miRNAs were *miR-199a*, four members of *miR-320* family and *miR-483*. *miR-199a* was described as involved in the maintenance of cell size in cardiomyocytes [28], and as a master regulator of a hypoxia-triggered pathway [29]. *miR-320* was shown to be involved in the regulation of cardiac ischemia/reperpusion injury through targeting heat-shock protein 20: over-expression enhanced cardiomyocyte apoptosis, whereas knockdown was cytoprotective [30]. *miR-483* was described as *in vitro* regulator of angiogenesis through serum response factor [31].

Further analysis using miRBase revealed that 43 differentially expressed miRNAs belong to 25 miRNA Gene family, and that half of differentially expressed miRNAs is clustered. Further, the 4 clusters are fully represented among differentially expressed miRNAs. This is expected since many genetically clustered and co-transcribed miRNAs are often expressed at different levels [26]. TAM tool was used to annotate 43 differentially expressed miRNAs; more than half of the defined functions are related to heart pathology and physiology, as well as to 21 disease outcomes related to heart pathology. Based on annotation we used nine miRNAs to validate microarray results, using two different qPCR technologies.

Most of the validated miRNAs have been already described as being involved in post-infarct remodelling [32]. Using TaqMan based technology; the *miR-1* and *miR-133* were used to compare present study to our-previous research [33] and confirmed up-regulation of *miR-1* in remote myocardium. However, to the best of our knowledge, this is the first report of *miR-98* down-regulation in human MI, which has been shown to be involved in regulation of cardiac hypertrophy [34], and its role in MI was proposed also through inflammation, although the verification still lacks [35]. Using Sybr Green the differential expression was shown for *miR-21*, which is deregulated in numerous cardiac diseases [24,36-38], although the role of *miR-21* in cardiac pathology is controversial at present. *miR-21* is broadly expressed in multiple tissue, expression in the heart cells is developmental and age-dependent [36], there is difference in expression between infracted regions and border zone [36] as well as in cell type (cardiomyocytes, fibroblasts, etc.) [39]. We also showed down-regulation of *miR-126*, which plays an important role in ischemic angiogenesis [40], *miR-125a/b*, which are involved (according to TAM) in myocardial remodelling after MI, although their target genes in cardiovascular pathology are not known at the present.

In addition, we showed that the expression analysis using TaqMan or Sybr Green gave similar results. We also showed that analysis of RNA*later* and FFPE with TaqMan gives the same results, which was also partially examined in our previous study [33]. In addition, analysis of RNA*later* and FFPE with Sybr Green is comparable. However, some discrepancies can be seen between RNA*later* and FFPE samples, and this can be due to sampling error. FFPE tissues are microscopically examined before cutting for subsequent RNA isolation, but this cannot be performed when using RNA*later* stored samples. Further, normalization strategy in qPCR experiments (often used for validation of microarray results) is in addition to the recent study [41] also important for the outcome of miRNA expression analysis in the human MI. Based on our experience, the best RG would be *RNU6B*, especially when using Sybr Green. In accordance to our previous studies [11,33], *miR-26b* is as good RG, but only when using TaqMan technology.

## Conclusion

It has been recently shown, that miRNAs are important regulators of calcium handling subunits, and might be also involved in regulation of calcium pumps [42,43]. Moreover, a correlation between SERCA2 expression and miRNAs has been demonstrated [44], and in accordance to that study is also our observation that more predicted binding sites are in SERCA2b than in SERCA2a. Although is hard to predict, which miRNA is involved in SERCA2 regulation, since the differentially expressed miRNA can be also from non-cardiomyocytes, we identified some good candidate miRNAs, which could be involved in the SERCA2 regulation (*miR-199a* for SERCA2b, *miR-140* for both isoforms, and *miR-574* for SERCA2a). The best candidate is *miR-574-3p*, which was also predicted to target SERCA2 in our previous study [11]. In contrast to SERCA2, which was down-regulated, both *miR-574-3p* and *-5p* showed up-regulation in infarcted tissue compared to corresponding remote myocardium as well as to healthy human hearts. There was also high difference in free-energy of binding and flanking region; seed region is also predicted to be involved in pairing. However, these are theoretical results based on expression patterns of miRNAs and SERCA2 and computational algorithms, which need to be further confirmed *in vitro* and/or *in vivo*[45]. These results could be a starting point for us and others for further validation and verification experiments regarding miRNAs and SERCA2 interaction.

In addition, we showed that the expression analysis using TaqMan or Sybr Green gave similar results. We also showed that analysis of RNA*later* and FFPE using either technology is comparable. Finally, we defined that normalization strategy is important for the outcome of miRNA expression analysis in the human MI.

## Methods

### Patients and tissue samples

Our study included autopsy samples of infarcted heart tissue and border zone, and the corresponding remote myocardium from 6 patients with myocardial infarction (MI), who died one week after MI. All autopsies were performed within 24 h after death. MI was diagnosed clinically by symptoms and/or electrocardiographic changes, and confirmed by elevated plasma levels of markers of cardiac necrosis. The duration of MI at the time of death was estimated on the basis of histological changes and clinical data. The rupture of free-wall was observed in 2 patients. Among patients with MI, there were 3 males and 3 females, aged 56–83 years (71.83 ± 10.83). Diabetes and arterial hypertension were recorded in 3 and 3 patients, respectively. Three patients had received reperfusion treatment and 2 had documented arrhythmias.

For all six patients with MI the paired tissue samples were from heart ventricle (infarcted tissue and remote myocardium) and were immediately stored in *RNAlater* (Ambion) as well as formalin-fixed paraffin-embedded (FFPE).

The investigation conforms to the principles outlined in the Helsinki Declaration. The Ethics Review Board of the National Medical Ethics Committee (NMEC) of the Republic of Slovenia granted approval for this research (39/01/08).

### Protein extraction

*RNAlater* stored tissue samples were homogenized in RIPA buffer (50 mM Tris HCl (Merck), pH 8; 150 mM NaCl (Merck); 1% (v/v) NP40 (USB); 0.5% sodium deoxycholate (Merck); 0.1% SDS (Sigma)) containing protease inhibitors in a final concentration of 1 mM EDTA (Calbiochem), pH 8, 1 mM sodium orthovanadate (Santa Cruz), 10 mM aprotinin (Sigma), 10 μg/ml leupeptin (Sigma), 10 μg/ml antiapin (Sigma), 1 μg/ml pepstatin (Sigma), 1 mM PMSF (Fluka). Homogenization of tissue was performed by Polytron PT3000 (Kinematica AG), followed by incubation for 30 min on ice and centrifugation at 10.000xg and 4°C for 15 min. The supernatant was transferred and stored at −70°C. The concentration of proteins in the tissue homogenate was measured by the BCA method (Nanodrop-1000, Thermo Scientific) using the BCA protein assay (Pierce) according to the manufacturer’s protocol.

### Western blot

Twenty μg of proteins of tissue homogenate was electrophoresed at 150 V and 4°C for 40 min through SDS-polyacrilamide gel (10% Tris-HEPES-SDS, Pierce). After electrophoresis was completed and equilibration of gel had been carried out, the proteins were electroblotted onto a 0.45 μm nitrocellulose membrane (Pierce) for 1.5 h at 40 V and 4°C. The membrane was blocked with 5% (w/v) blocking reagent (Amersham) in TTBS buffer (20 mM Tris (Merck), 150 mM NaCl (Merck), pH 7.4, 0.05% (v/v) Tween-20 (Sigma)) overnight at 4°C. After blocking, the membrane was incubated with mouse anti-SERCA2 (Santa Cruz, sc-73022) for 2 h at room temperature, diluted 1:100 in 5% blocking reagent (Amersham) in TTBS buffer, and mouse anti-GAPDH (Santa Cruz, sc-69778) for 2 h at room temperature, diluted 1:300 in 5% blocking reagent (Amersham) in TTBS buffer. GAPDH was used as internal control gene. Three washes for 5 min each time were performed in TTBS buffer and the membrane was incubated with horseradish peroxidase-conjugated anti-mouse (Santa Cruz Biotehnology) for 2 h at room temperature, diluted 1:2000 in 5% blocking reagent (Amersham) in TTBS buffer. After three more washes, ECL detection by ECL Western Blotting Substrate (Pierce) and Hyperfilm ECL (Amersham) was performed according to the manufacturer’s protocol.

### miRNA isolation and analysis of extracted miRNA

#### RNA isolation from RNA*later* stored tissue samples

Autopsy samples stored in *RNAlater* (Ambion) were used for RNA extraction according to manufacturer protocol. Total RNA isolation was performed using a miRNeasy kit (Qiagen) according to the manufacturer’s protocol. All the reagents were from Qiagen, except where otherwise indicated. Briefly, the tissue was homogenized by Polytron PT3000 (Kinematica AG) in 1 ml of QIAzol Lysis Reagent, following addition of 200 μl of chloroform and centrifugation for 15 min at 12.000xg. After addition of 100% ethanol (Merck, USA) to the aqueous phase the mixture was transferred to an RNeasy Mini spin column. Three washing steps were further performed (using buffers RWT and RPE) as well as on-column DNase digestion (RNase-Free DNase Set). The RNA was eluted in 50 μl of nuclease-free water.

#### RNA isolation from FFPE tissue samples

Three to eight 10-μm sections were used for the isolation procedure. Total RNA isolation was performed using a miRNeasy FFPE kit (Qiagen) according to the manufacturer’s protocol. All the reagents were from Qiagen, except where otherwise indicated. Briefly, 1 ml of Xylene (Merck) was added for de-paraffinization, followed by brief vortexing and centrifugation. After the ethanol-washing step (using 1 ml of 100% ethanol), pellets were air-dried and digestion with Proteinase K in 0.25 ml digestion mix was performed at 55°C for 15 min. Further, 15 min incubation at 80°C in order to partially reverse formaldehyde modification of nucleic acid was performed. After adding 0.5 ml of RBC buffer and gDNA elimination step, 1.75 ml of 100% ethanol (Merck) was added to the eluted samples and the mixture was transferred to an RNeasy MiniElute spin column. After two washing steps (using buffer RPE), the RNA was eluted in 30 μl of nuclease-free water.

#### Concentration and integrity measurements of extracted RNA

The concentration of the extracted RNA was measured using NanoDrop-1000 (Thermo Scientific) and tested for UV/vis ratios. The A_260_/A_230_ nm intensity ratio needs to be above 1.0 and A_260_/A_280_ ratio needs to be above 1.8. The integrity and presence of small RNAs (<200 nucleotides) was analyzed on a Bioanalyzer 2100 (Agilent) using the high resolution Small RNA Assay.

### microRNA microarray analysis

Expression analysis was performed using hybridization to μParaflo® microfluidics micorarrays (LC Sciences) in the Sanger miRBase database Release 10.1 (719 predicted mature human miRNA probes, MRA-1001), and spike-in/perfectly matched and mismatched probes for quality control. Each miRNA probe was represented 5 times on a single microarray. The control probes were spiked into the RNA samples before labelling. Five to ten μg of RNA from heart samples was used for miRNA microarray. Using size-fractionation, small RNA (<300 nt) enrichment was performed, following extension with a poly(A) tail, to which the an oligonucleotide tag was added for subsequent labelling. The two sets of RNA sequences were labelled with different affinity tags to allow simultaneous detection of two samples using dual colour labelling (Cy3 and Cy5) and dye-reversal. Three microRNA microarrays were performed, using RNA samples from 3 infarcted tissues and compared to 3 corresponding remote myocardium.

To detect small amounts of miRNA, standard data analysis was performed (calculation of signal intensities, determination of detectable transcripts, and calculation of differential ratios). The signal was amplified and the background was subtracted using the local regression method. Spot signals normalization was carried out using a cyclic LOWESS (Locally-weighted regression) method (resulting in so-called processed data). The miRNAs were treated as detected, when the signals of the repeating probes were above detection level in at least 3 of 5 probes for each miRNA. For the dual colour experiment, the ratio of the two sets of detected signals (Log2 transformed, balanced) and p-values of the *t*-test were calculated; differentially detected signals were those with less than 0.01 of p-values. All data processes are carried out using in-house developed computer programs by LC sciences. Data adjustment includes data filtering, Log2 transformation, and gene centring and normalization. miRNAs with intensity values below a threshold value (data filtering) were removed and log_2_ transformation was used to convert expression values into a linear scale for statistical comparison. Gene centring and normalization transform the Log2 values using the mean and the standard deviation of individual genes across all samples.

Further microarray analysis using processed data and the Acuity 4.0 program defined which miRNAs were differently expressed in all of the microarrays performed (infarcted tissue compared to corresponding remote myocardium). MiRNA expression heat map was constructed by unsupervised hierarchical clustering to reveal the relationship between differentially expressed miRNAs.

### Bioinformatics analysis and miRNA prediction

#### SERCA2:miRNA prediction

To investigate SERCA2 mRNA as a target for miRNA regulation, 3'-UTRs mRNA sequences of corresponding SERCA2a and SERCA2b were analyzed by various computational algorithms, which utilize distinct parameters to predict the probability of a functional miRNA-binding site within a given mRNA target. We used the following bioinformatic algorithms to predict miRNA target sites: PicTar [13], TargetScan5.1 [12], mirTarget2 included in miRDB [14], and miRanda [15] included in databases miRBase (MicroCosm Targets) [16] and in microrna.org [17].

#### SERCA2a:miRNA free-energy binding analysis

To further investigate the putative miRNAs (up-regulated miRNAs in infracted tissue) that may repress the expression of mRNA SERCA2, we performed *in silico* free-energy validation. We determined the free-energy (ΔG) of the miRNA:mRNA binding, using the RNA22 algorithm [19], and ΔG 70 nt flanking the 5' and 3' sides of the predicted miRNA binding sites, using RNAfold [20]. According to Zhao et al. (2005) [18] we tested the following presumptions to verify the miRNA as potential regulator of SERCA2: (i) if miRNAs seed regions are involved in binding; (ii) we calculated the free-energy of binding SERCA2a:miRNAs and 70 nt flanking the 5' and 3' sides. Sequences for 3-UTR SERCA2a and SERCA2b were downloaded from NCBI, accession numbers NM_001681.3 and NM_170665.3, respectively, and from Ensembl, transcript ID ENST00000313432 and ENST00000308664, respectively.

#### miRNA annotation and functional classification

Using miRBase [16] the differentially expressed miRNAs were searched for genomic location, clusters and miRNA Gene family. TAM tool [21] was used to annotate differentially expressed miRNAs in the mean of function and disease associations, and miRNApath [22] was used to search for miRNAs that may be involved in calcium signalling pathway, and could regulate two other proteins that regulate SERCA2 expression, phosphlamban (PLN) and sarcolipin (SLN).

### Quantitative real-time PCR (qPCR)

Some miRNAs differentially expressed in miRNA microarray expression analysis were validated using the miScript system (Qiagen) or TaqMan based technology (Applied Biosystems). All the reagents were from Qiagen or Applied Biosystems, respectively, except where otherwise indicated. qPCR was carried out using the Rotor Gene Q (Qiagen) or ABI 7900 Real-Time PCR System (Applied Biosystems).

Prior to qPCR analysis two pools of RNA samples were created, obtained from RNA*later* stored and FFPE tissue samples. After reverse transcription, the cDNA was diluted in five steps, ranging from 3-fold dilution to 243-fold dilution, and the probes were tested for PCR efficiency. All the qPCR efficiency reactions were performed in triplicate.

#### miScript System (Qiagen)

No-reverse transcriptase (no-RT) control included all the qPCR reagents without the reverse transcription (RT) step. If the product was amplified, it indicated that there was still some genomic DNA contamination. Using DNA digestion step, the RNA obtained from heart tissue samples was treated with DNAse I (RNase-Free DNase Set, Qiagen) prior to qPCR, if the product was amplified during no-RT control. Up to 1 μg (5 μl) of RNA contaminated with genomic DNA was used, adding 2 μl 10x DNase buffer (RDD buffer), 10 units RNase inhibitor, 0.5 Kunitz units DNase I and RNase-free water to obtained 20 μl reaction mixtures. After 30 min incubation step at 37°C, 2 μl 140 mM EDTA (Sigma) was added and incubate for 5 min at 65°C to inactivate DNase I. Concentration was measured again using NanoDrop-1000. An miScript reverse transcription kit was used for RT. Briefly, a 15 μl reaction master mix was created, containing 100 ng of total RNA, 3 μl 5x miScript RT buffer, 0.75 μl miScript reverse transcriptase mix and 10 units (0.33 μl) RNase inhibitor. After incubation for 60 min at 37°C and 5 min at 95°C, the cDNA was diluted 10-fold, and 3 μl was used for each qPCR reaction. Ten μl PCR master mix contained 5 μl 2x QuantiTect SYBR Green PCR Master Mix, 1 μl 10x miScript universal primer and 1 μl 10x miScript Primer Assay. All the qPCR reactions were performed in duplicate as following: initial denaturation at 95°C for 15 min, and 40 cycles for 15 s at 94°C (denaturation), for 30 s at 55°C (primers annealing), and for 30 s at 70°C (elongation). Following amplification, melting curves were acquired on the SYBR channel using a ramping rate of 1°C/60 s for 60–95°C. MicroRNAs, *miR-122, miR-125a-5p, miR-125b, miR-126, miR-21* were tested relatively to *RNU6B* and *miR-26b*. *RNU6B* and *miR-26b* were tested as the RGs. The signal was collected at the endpoint of every cycle.

#### TaqMan based technology

Looped primers for specific miRNA RT were utilized following the manufacturer’s protocol. *RNU6B*, *RNU48* and *miR-26b* were used as RGs, according to the manufacturer’s protocol. MicroRNAs, *miR-1, miR-133a, miR-133b,* and *miR-98* were tested relatively to *RNU6B, RNU48* and *miR-26b*. Briefly, the 10 μl RT reaction master mix was performed with 10 ng of total RNA sample, 0.71 μl of MultiScribe Reverse Transcriptase (50 U/μl), 1 μl of Reverse Transcription Buffer (10x), 0.1 μl of dNTP (100 mM), 0.19 μl RNAase inhibitor (20 U/μl), and 2 μl of RT primer (5x). The reaction conditions were: at 16°C for 30 min, at 42°C for 30 min, at 85°C for 5 min. qPCR was carried out in 20 μl PCR master mix containing 10 μl TaqMan 2x Universal PCR Master Mix, 1 μl TaqMan assay, 9 μl RT products diluted 7-fold. The qPCR reactions were performed in duplicates or triplicates as following: initial denaturation at 95°C for 10 min, and 40 cycles for 15 s at 95°C (denaturation), for 60 s at 60°C (primers annealing and elongation). The signal was collected at the endpoint of every cycle.

#### Statistical analysis

For miRNA expression analysis using qPCR, the calculation based on efficiency correction was used [46]. Statistical analysis was performed using nonparametric Willcoxon Rank test and Spermans Rho correlation coefficient, both of which are included in SPSS statistical software (v20), with a cut off point at p < 0.05. When the differences in ratio of tested groups reached or were below p = 0.05, the difference in miRNA expression was considered as statistical significant.

## Abbreviations

BCA: Bicinchoninic acid; ECL: Enhanced chemiluminiscence; FFPE: Formalin-fixed paraffin-embedded; ΔG: Free-energy; GAPDH: Glyceraldehyde-3-phosphate dehydrogenase; miRNA: microRNA; mRNA: Messenger RNA; MI: Myocardial infarction; nt: Nucleotide; PLN: Phospholamban; qPCR: Quantitative PCR; RG: Reference gene; RIPA: Radioimmunoprecipitation assay; RT: Reverse transcription; SERCA2: Sarcoplasimic reticulum calcium ATPase; SLN: Sarcolipin; TTBS: Tris-tween buffered saline; UTR: Untranslated region.

## Competing interests

The authors declare that they have no competing interests.

## Authors’ contributions

EB participated in the design of the study, analysis and interpretation of data; NZ estimated the duration of myocardial infarction at the time of death on the basis of histological changes and clinical data; DG have been involved in drafting the manuscript, revising it critically and have given final approval of the version to be published. All authors read and approved the final manuscript.

## Supplementary Material

Additional file 1 **Table S1.** Predicted miRNAs to target SERCA2 according to the used programs. Click here for file

Additional file 2 ** Table S2.** Annotation of differentially expressed miRNAs using TAM tool – functions. Click here for file

Additional file 3 **Table S3. **Annotation of differentially expressed miRNAs using TAM tool - disease association (HMDD) related to heart diseases, development and physiology. Click here for file
