# Supplementary material for: MicroRNAs and cardiac sarcoplasmic reticulum calcium ATPase-2 in human myocardial infarction: expression and bioinformatic analysis
Source: BMC Genomics. 2012 Oct 15;13:552. doi: 10.1186/1471-2164-13-552 (PMC3532181; doi:10.1186/1471-2164-13-552)
Supplement: Additional file 1 — Table S1. Predicted miRNAs to target SERCA2 according to the used programs. [file 1471-2164-13-552-S1.docx]

**Additional file 1: Table S1**

**Table of predicted miRNAs to target SERCA2 according to the used programs**

Legend: 2a, an isoform SERCA2a; 2b, an isoform SERCA2b.

| Prediction algorithm | Target Scan | PicTar | miRTarget2 | miRanda | |
| --- | --- | --- | --- | --- | --- |
| Database |  | | miRDB | microRNA.org | MicroCosm  (miRbase) |
| miRNAs |  | |  |  |  |
| hsa-let-7a |  | 2b |  | 2b | 2b |
| hsa-let-7b |  | 2b |  | 2b | 2b |
| hsa-let-7c |  | 2b |  | 2b | 2b |
| hsa-let-7d |  | 2b |  | 2b | 2b |
| hsa-let-7e |  | 2b |  | 2b | 2b |
| hsa-let-7f |  | 2b |  | 2b | 2b |
| hsa-let-7g |  | 2b |  | 2b | 2a, 2b |
| hsa-let-7g* |  |  |  |  | 2a |
| hsa-let-7i |  | 2b |  | 2b | 2b |
| hsa-let-7i* |  |  |  |  | 2b |
| hsa-miR-20a* |  |  |  |  | 2a |
| hsa-miR-22 |  |  |  | 2b |  |
| hsa-miR-24 |  | 2b |  | 2b |  |
| hsa-miR-25 | 2a | 2a | 2a | 2a | 2a |
| hsa-miR-28-3p |  |  |  | 2b | 2b |
| hsa-miR-29a* |  |  |  |  | 2a |
| hsa-miR-29b-1* |  |  |  |  | 2a |
| hsa-miR-30d |  | 2b |  | 2b | 2b |
| hsa-miR-30a |  |  |  | 2b | 2b |
| hsa-miR-30a-5p |  | 2b |  |  |  |
| hsa-miR-30e-5p |  | 2b |  |  |  |
| hsa-miR-30b |  | 2b |  | 2b |  |
| hsa-miR-30c |  | 2b |  | 2b | 2b |
| hsa-miR-30e |  | 2b |  | 2b | 2b |
| hsa-miR-31 |  |  |  | 2b |  |
| hsa-miR-32 | 2a | 2a | 2a | 2a |  |
| hsa-miR-33a |  |  |  | 2a |  |
| hsa-miR-33b |  |  |  |  | 2a |
| hsa-miR-34a |  |  |  | 2b |  |
| hsa-miR-34c |  |  |  |  | 2b |
| hsa-miR-92 |  | 2a |  |  | 2a |
| hsa-miR-92a | 2a |  | 2b | 2a | 2a |
| hsa-miR-92b | 2a |  | 2a | 2a | 2a |
| hsa-miR-96 |  |  |  |  | 2b |
| hsa-miR-98 |  | 2b |  | 2b | 2b |
| hsa-miR-103 | 2a |  |  |  |  |
| hsa-miR-107 | 2a |  |  |  |  |
| hsa-miR-130a |  | 2b |  |  |  |
| hsa-miR-130b |  | 2b |  |  |  |
| hsa-miR-132 |  |  |  | 2a |  |
| hsa-miR-135a |  |  |  | 2a |  |
| hsa-miR-135b |  |  |  | 2a |  |
| hsa-miR-141 |  | 2b |  | 2b |  |
| hsa-miR-142-3p | 2a | 2a | 2a |  | 2a |
| hsa-miR-148b |  | 2b |  | 2b | 2b |
| hsa-miR-148a |  | 2b |  | 2b |  |
| hsa-miR-149 | 2a | 2b | 2a | 2b |  |
| hsa-miR-151-3p |  |  | 2a | 2b |  |
| hsa-miR-152 |  | 2b |  | 2b | 2b |
| hsa-miR-155* |  |  |  |  | 2a |
| hsa-miR-181a |  | 2b | 2a | 2b | 2a |
| hsa-miR-181b |  | 2b | 2a | 2b | 2b |
| hsa-miR-181c |  | 2b | 2a | 2b |  |
| hsa-miR-181d |  |  | 2a | 2b |  |
| hsa-miR-182 |  | 2b |  | 2b |  |
| hsa-miR-185 | 2a | 2a | 2a |  |  |
| hsa-miR-186 |  | 2a |  |  |  |
| hsa-miR-192 |  |  |  |  | 2b |
| hsa-miR-192* |  |  |  |  | 2b |
| hsa-miR-195 |  |  |  | 2a, 2b |  |
| hsa-miR-200a |  | 2b |  | 2b |  |
| hsa-miR-200b | 2a |  | 2a | 2b | 2b |
| hsa-miR-200c | 2a | 2b | 2a | 2b | 2b |
| hsa-miR-205 |  |  |  | 2b |  |
| hsa-miR-212 |  |  |  | 2a |  |
| hsa-miR-214 |  |  |  | 2b | 2b |
| hsa-miR-219 | 2a |  |  |  |  |
| hsa-miR-220c | 2a |  | 2a | 2a |  |
| hsa-miR-293 |  |  |  |  | 2b |
| hsa-miR-296-3p | 2a |  |  | 2a |  |
| hsa-miR-298 |  |  |  | 2b | 2b |
| hsa-miR-299 | 2a |  |  |  |  |
| hsa-miR-300 |  |  | 2a | 2b |  |
| hsa-miR-301 |  | 2b |  |  |  |
| hsa-miR-320a |  |  |  | 2b |  |
| hsa-miR-320b |  |  |  | 2b |  |
| hsa-miR-326 | 2a |  |  |  |  |
| hsa-miR-330 | 2a |  |  |  |  |
| hsa-miR-338-3p |  |  |  | 2a |  |
| hsa-miR-338-5p | 2a |  | 2a | 2a |  |
| hsa-miR-340 | 2a |  |  | 2b |  |
| hsa-miR-341 |  |  |  |  | 2b |
| hsa-miR-345 |  | 2b |  |  |  |
| hsa-miR-346 | 2a | 2b |  |  |  |
| hsa-miR-363 | 2a |  | 2a | 2a | 2a |
| hsa-miR-367 | 2a | 2a | 2a | 2a | 2a |
| hsa-miR-369-3p |  |  |  | 2b |  |
| hsa-miR-374b |  |  |  |  | 2b |
| hsa-miR-376a | 2a |  |  | 2a |  |
| hsa-miR-376b | 2a |  |  | 2a |  |
| hsa-miR-379 | 2a |  |  |  |  |
| hsa-miR-381 |  |  | 2a | 2b | 2b |
| hsa-miR-384 |  |  |  | 2b | 2b |
| hsa-miR-409-3p |  |  |  | 2b |  |
| hsa-miR-410 | 2a |  |  | 2a | 2a |
| hsa-miR-421 | 2a |  |  |  |  |
| hsa-miR-429 | 2a |  | 2a | 2b | 2b |
| hsa-miR-450b-5p | 2a |  |  | 2a |  |
| hsa-miR-452 |  |  |  |  | 2b |
| hsa-miR-455-5p |  |  |  | 2b |  |
| hsa-miR-487a | 2a |  |  |  |  |
| hsa-miR-489 | 2a |  |  |  |  |
| hsa-miR-491-5p |  |  |  | 2a |  |
| hsa-miR-493* |  |  |  |  | 2b |
| hsa-miR-495 | 2a |  |  | 2a |  |
| hsa-miR-496 |  |  | 2a | 2b | 2b |
| hsa-miR-499-3p |  |  |  | 2b | 2b |
| hsa-miR-499-5p |  |  |  | 2b? | 2b? |
| hsa-miR-507 | 2a |  |  |  |  |
| hsa-miR-508-5p |  |  | 2a |  |  |
| hsa-miR-516b | 2a |  | 2a | 2a, 2b |  |
| hsa-miR-517b |  |  |  |  | 2a, 2b |
| hsa-miR-518-5p |  |  |  |  | 2b |
| hsa-miR-518c |  |  |  |  | 2b |
| hsa-miR-518c* |  |  |  |  | 2a |
| hsa-miR-518d-5p |  |  |  | 2b |  |
| hsa-miR-519 |  |  |  | 2b |  |
| hsa-miR-519b-5p |  |  |  | 2b |  |
| hsa-miR-520c-5p |  |  |  | 2b |  |
| hsa-miR-520g | 2a |  |  | 2a, 2b |  |
| hsa-miR-520h | 2a |  |  | 2a, 2b |  |
| hsa-miR-522 | 2a |  |  | 2a |  |
| hsa-miR-523 |  |  |  |  | 2a, 2b |
| hsa-miR-526a |  |  |  | 2b |  |
| hsa-miR-539 | 2a |  |  | 2a |  |
| hsa-miR-544 | 2a |  | 2a | 2a, 2b |  |
| hsa-miR-545 | 2a |  |  | 2a |  |
| hsa-miR-548a-3p |  |  |  | 2b |  |
| hsa-miR-548c-3p |  |  |  | 2b |  |
| hsa-miR-548d | 2a |  | 2a | 2b |  |
| hsa-miR-548e |  |  |  | 2b |  |
| hsa-miR-548f |  |  |  | 2b |  |
| hsa-miR-548g | 2a |  | 2a | 2b |  |
| hsa-miR-548i |  |  |  | 2b |  |
| hsa-miR-548k |  |  |  | 2b |  |
| hsa-miR-548n | 2a |  |  | 2b |  |
| hsa-miR-548p |  |  |  | 2b |  |
| hsa-miR-555 |  |  |  | 2b |  |
| hsa-miR-557 | 2a |  |  | 2b |  |
| hsa-miR-560 |  |  |  |  | 2a |
| hsa-miR-562 | 2a |  | 2a | 2b | 2a |
| hsa-miR-567 |  |  |  | 2b | 2b |
| hsa-miR-568 |  |  | 2a | 2b |  |
| hsa-miR-571 | 2a |  | 2a | 2a |  |
| hsa-miR-574-3p | 2a |  |  | 2a | 2a |
| hsa-miR-575 |  |  |  | 2b |  |
| hsa-miR-576-3p | 2a |  | 2a | 2a |  |
| hsa-miR-577 | 2a |  |  | 2a |  |
| hsa-miR-582-3p |  |  |  | 2b | 2b |
| hsa-miR-582-5p | 2a |  |  | 2b |  |
| hsa-miR-583 |  |  |  | 2b |  |
| hsa-miR-586 | 2a |  | 2a | 2a, 2b |  |
| hsa-miR-590-5p |  |  |  | 2b | 2b |
| hsa-miR-593 |  |  |  | 2a |  |
| hsa-miR-603 |  |  | 2a |  |  |
| hsa-miR-608 |  |  |  | 2b |  |
| hsa-miR-610 |  |  | 2a | 2a |  |
| hsa-miR-612 |  |  | 2a |  |  |
| hsa-miR-619 |  |  |  | 2a | 2a |
| hsa-miR-626 | 2a |  |  |  | 2a |
| hsa-miR-628-5p |  |  | 2a |  | 2b |
| hsa-miR-632 | 2a |  |  | 2a, 2b | 2b |
| hsa-miR-634 | 2a |  | 2a | 2a |  |
| hsa-miR-647 | 2a |  |  |  |  |
| hsa-miR-650 | 2a |  |  |  |  |
| hsa-miR-651 | 2a |  |  | 2a |  |
| hsa-miR-654 | 2a |  |  | 2b |  |
| hsa-miR-654-3p |  |  | 2a | 2a, 2b | 2b |
| hsa-miR-655 | 2a |  | 2a | 2a |  |
| hsa-miR-658 |  |  |  | 2b |  |
| hsa-miR-663 |  |  |  | 2b |  |
| hsa-miR-664 |  |  | 2a | 2b | 2b |
| hsa-miR-668 | 2a |  |  |  |  |
| hsa-miR-671-5p |  |  |  | 2b |  |
| hsa-miR-674 |  |  |  |  | 2b |
| hsa-miR-679 |  |  |  |  | 2b |
| hsa-miR-708* |  |  |  |  | 2b |
| hsa-miR-743b-3p |  |  |  |  | 2b |
| hsa-miR-744 |  |  |  | 2b |  |
| hsa-miR-760 |  |  |  |  |  |
| hsa-miR-764-3p |  |  |  |  | 2b |
| hsa-miR-767 |  |  | 2a | 2b |  |
| hsa-miR-768-3p | 2a |  |  | 2b |  |
| hsa-miR-805 |  |  |  |  | 2b |
| hsa-miR-875-5p |  |  |  | 2b | 2b |
| hsa-miR-876-3P |  |  | 2a |  |  |
| hsa-miR-882 | 2a |  |  |  |  |
| hsa-miR-887 |  |  |  | 2b |  |
| hsa-miR-879 |  |  |  | 2a |  |
| hsa-miR-922 |  |  | 2a | 2a, 2b | 2b |
| hsa-miR-923 |  |  |  | 2b |  |
| hsa-miR-934 | 2a |  | 2a | 2a | 2a |
| hsa-miR-942 | 2a |  |  | 2a, 2b |  |
| hsa-miR-1179 | 2a |  |  |  |  |
| hsa-miR-1183 | 2a |  |  |  |  |
| hsa-miR-1192 | 2a |  |  |  |  |
| hsa-miR-1206 |  |  | 2a | 2a |  |
| hsa-miR-1226 |  |  | 2a | 2a |  |
| hsa-miR-1227 |  |  |  | 2a |  |
| hsa-miR-1238 | 2a |  |  |  |  |
| hsa-miR-1248 |  |  |  | 2a |  |
| hsa-miR-1252 |  |  |  | 2b |  |
| hsa-miR-1253 |  |  |  | 2a |  |
| hsa-miR-1255a |  |  | 2a | 2a |  |
| hsa-miR-1255b |  |  |  | 2a |  |
| hsa-miR-1261 |  |  |  | 2a, 2b |  |
| hsa-miR-1262 |  |  |  | 2a |  |
| hsa-miR-1273 | 2a |  |  |  |  |
| hsa-miR-1283 |  |  | 2a | 2a |  |
| hsa-miR-1284 |  |  | 2a |  |  |
| hsa-miR-1285 |  |  | 2a |  |  |
| hsa-miR-1299 |  |  |  | 2a |  |
| hsa-miR-1301 | 2a |  |  |  |  |
