# Supplementary material for: MicroRNAs and cardiac sarcoplasmic reticulum calcium ATPase-2 in human myocardial infarction: expression and bioinformatic analysis
Source: BMC Genomics. 2012 Oct 15;13:552. doi: 10.1186/1471-2164-13-552 (PMC3532181; doi:10.1186/1471-2164-13-552)
Supplement: Additional file 2 — Table S2. Annotation of differentially expressed miRNAs using TAM tool – functions. [file 1471-2164-13-552-S2.docx]

**Additional file 2: Table S2 Annotation of differentially expressed miRNAs using TAM tool –** **functions.**

| Term | Count | Percent | microRNAs differentially expressed |
| --- | --- | --- | --- |
| Activation of caspases cascade | 2 | 0.33 | let-7, miR-150 |
| Akt pathway | 2 | 0.12 | miR-26b, miR-19b |
| Angiogenesis | 10 | 0.42 | let-7b, miR-21, miR-26, miR-320, miR-16, miR-122, miR-19b, miR-378, miR-27b, miR-150 |
| Apoptosis | 11 | 0.25 |  |
| Cardiogenesis | 2 | 0.67 | miR-1, miR-133a |
| Cell differentiation | 5 | 0.29 | miR-1, miR-150, miR-145, miR-143, miR-16 |
| Cell proliferation | 11 | 0.36 | let-7c, let-7d, miR-125b, miR-21, miR-16, miR-150, miR-27b, miR-145, miR-143, miR-140, miR-19b |
| Epithelial-mesenchymal transition | 12 | 0.29 | let-7b, let-7c, let-7d, miR-21, miR-125a, miR-30a/e/b/c/d, miR-29a/c |
| Granulopoiesis | 3 | 0.30 | let-7c, let-7d, miR-21 |
| Inflammation | 13 | 0.32 | miR-1, miR-320, miR-125b, let-7g, miR-126, miR-150, miR-133a, miR-199a, miR-98, miR-27b, miR-143, miR-140, miR-21 |
| Muscle development | 5 | 0.45 | miR-1, miR-133a, miR-499, miR-23a, miR-133b |
| Cell division | 6 | 0.35 | let-7b, let-7g, let-7c, let-7d, miR-16, miR-27a |
| Cell fate determination | 5 | 0.19 | let-7b, let-7c, let-7g, let-7d, miR-150 |
| Cell motility | 3 | 0.21 | miR-145, miR-143, miR-21 |
| Glucose metabolism | 1 | 0.17 | miR-23a |
| Heart development | 6 | 0.86 | miR-1a, miR-499, miR-133a/b, miR-27b, miR-21 |
| Hematopoiesis | 12 | 0.39 | miR-1, miR-125b, miR-126, miR-378, miR-133a, miR-19b, miR-150, miR-145, miR-143, miR-29c, miR-98, miR-29a |
| Immune system | 2 | 0.11 | miR-150, miR-19b |
| Smooth muscle cell fate | 2 | 1 | miR-145, miR-143 |
