# Supplementary material for: MicroRNAs and cardiac sarcoplasmic reticulum calcium ATPase-2 in human myocardial infarction: expression and bioinformatic analysis
Source: BMC Genomics. 2012 Oct 15;13:552. doi: 10.1186/1471-2164-13-552 (PMC3532181; doi:10.1186/1471-2164-13-552)
Supplement: Additional file 3 — Table S3. Annotation of differentially expressed miRNAs using TAM tool - disease association (HMDD) related to heart diseases, development and physiology. [file 1471-2164-13-552-S3.docx]

**Additional file 3: Table S3 Annotation of differentially expressed miRNAs using TAM tool - disease association (HMDD) related to heart diseases, development and physiology.**

| Term | Count | Percent | microRNAs differentially expressed |
| --- | --- | --- | --- |
| Aortic valve insufficiency | 3 | 1 | miR-195, miR-26a, miR-30b |
| Aortic valve stenosis | 3 | 1 | miR-195, miR-26a, miR-30b |
| Arrhythmias, cardiac | 2 | 1 | miR-1, miR-133a |
| Atherosclerosis | 3 | 0.27 | miR-145, miR-21, miR-126 |
| Cardiomyopathy, dilated | 1 | 0.50 | miR-499 |
| Cardiomyopathy, hypertrophic | 9 | 0.64 | miR-1, miR-125b, miR-21, miR-133a, miR-23a, miR-199a, miR-150, miR-27b, miR-195 |
| Cardiovascular diseases | 1 | 0.50 | miR-21 |
| Coronary artery disease | 3 | 0.27 | miR-1, miR-21, miR-126 |
| Heart defect, congenital | 5 | 0.30 | miR-1, miR-195, miR-26a, miR-133a, miR-30b |
| Hypertension | 3 | 0.50 | miR-1, miR-133a, miR-21 |
| Hypertrophy | 9 | 0.53 | let-7b, let-7c, let-7g, let-7d, miR-1, miR-133a, miR-98, miR-26a, miR-21 |
| Hypertrophy, left ventricular | 4 | 1.00 | miR-30a, miR-30b, miR-133a, miR-133b |
| Inflammation | 2 | 0.17 | miR-21, miR-29a |
| Ischemia | 1 | 0.33 | miR-320a |
| Myocardial infarction | 6 | 0.40 | miR-1, miR-21, miR-126, miR-133a, miR-150, miR-499 |
| Myocardium | 2 | 0.50 | miR-21, miR-29a |
| Myocytes, cardiac | 4 | 0.80 | miR-199a, miR-133a, miR-21, miR-133a |
| Stroke | 2 | 0.33 | miR-122, miR-133a |
| Vascular Diseases | 4 | 0.50 | miR-145, miR-23a, miR-126, miR-21 |
| Wounds and Injuries | 2 | 1.00 | miR-145, miR-143 |
